# How dispositional optimism–pessimism relates to early adolescents’ emotional maladjustment during COVID-19? Moderating roles of knowledge about the disease and parent-child conflicts

**DOI:** 10.3389/fpsyt.2025.1470733

**Published:** 2025-02-20

**Authors:** Yongqiang Jiang, Dazhou Wu, Xiuyun Lin

**Affiliations:** ^1^ Faculty of Education, Beijing Normal University, Beijing, China; ^2^ Faculty of Psychology, Beijing Normal University, Beijing, China

**Keywords:** COVID-19, optimism-pessimism, emotional maladjustment, knowledge about the disease, parent-child conflicts

## Abstract

**Introduction:**

The COVID-19 pandemic and associated containment measures changed the daily lives of children and adolescents around the world. To investigate the individual differences in emotional maladjustment under the COVID-19 pandemic, this study focused on the roles of dispositional optimism-pessimism, knowledge about the COVID-19 disease, and conflicts with parents among Chinese early adolescents.

**Method:**

edge about the COVID-19 disease, and conflicts with parents among Chinese early adolescents. The participants were 2,958 early adolescents aged 10 to 14 years old who completed online questionnaires during the pandemic.

**Results:**

While higher pessimism and lower optimism both led to increased emotional maladjustment, pessimism made a greater contribution. Knowledge about the disease and parent-child conflicts were both risk factors for adolescents’ emotional maladjustment, yet optimism and pessimism interacted with different factors. More knowledge about the disease intensified the effect of pessimism, and more parent-conflict undermined the effect of optimism.

**Discussion:**

Our findings provide directions for future aid in adolescence during hard periods depending on one’s personality.

## 1 Introduction

Under the pressure of the COVID-19 pandemic, people, from young to old, suffered various extents of psychological pressure, such as panic, anxiety, depression, loss, and posttraumatic stress (1–4). Among the general population, early adolescents deserve special attention (5). In such a life phase, adolescents have strong desires to be free and connected with peers (6). However, because of the pandemic, school suspension, discouragement of outdoor activities, social isolation from peers, and distance education have disrupted their daily life routines (7). In most cases, their parents were at home during the pandemic, and had to deal with both their own working affairs and their children’s daily management (8). The routine disruption and staying at home under parents’ monitoring might have put adolescents at high risk of emotional maladjustment (3, 9, 10). Individual differences were always common, i.e., some were less vulnerable to the hard times. This study aimed to investigate, during the COVID-19 pandemic, how adolescents’ individual differences in emotional maladjustment were related to their dispositional optimism−pessimism.

### 1.1 Dispositional optimism−pessimism and emotional maladjustment

An optimist sees a half-filled glass as half full, while a pessimist thinks it’s half as empty. The universal folk saying describes two different ways that people evaluate about their problems, adversity, or resources, and expectancies for the future. Generally, it is conceptualized as dispositional optimism (11). The Life Orientation Test (LOT; 12) and the LOT-Revised are the two most widely used scales to measure optimism (13). Traditionally, optimism and pessimism are regarded as bipolar traits on the same scale (13). The negatively framed items (e.g., assessing the presence of pessimism) are recorded and then added to the positively framed items (e.g., assessing the presence of optimism) to produce an overall scale score of optimism (e.g., 14). However, some studies questioned this notion, stating that optimism and pessimism are two different dimensions (15, 16). Frist, optimism and pessimism are conceptually different. People who are low in pessimism do not have to be high in optimism. Second, from the empirical evidences, correlations between the optimism and pessimism subscales are modest (e.g., 17, 18). For example, For example, Scheier and colleagues (18) reported that the absence of pessimism was more predictive of physical health than was the presence of optimism. Third, in relation to the Big Five personality traits, genetic studies found that pessimism is associated with neuroticism, while optimism is associated with traits beyond neuroticism, including extraversion, conscientiousness, and agreeableness (19, 20). Another genetic study distinguished optimism and pessimism from the five personality traits, suggesting independence for both (21).

Optimism by definition is related to well-being, while pessimism is on the contrary (11). During the hard period of COVID-19, optimists may experience less maladjustment than pessimists (11, 22). Youth with high optimism are likely to have positive outcome expectancies (12). They tend to believe that the pandemic will be controlled effectively and that life will be righting soon (23). The positive expectations are free of negative emotional reactions (e.g., anger, panic, and anxiety). In contrast, people with high pessimism are likely to expect negative outcomes. They may believe that they must stay at home and take online classes under parents’ monitoring for a long time. Moreover, optimists prefer to engage in coping strategies (e.g., problem-solving) rather than disengagement strategies (e.g., avoidance) when under stress (24, 25). Previous studies have demonstrated the positive associations of dispositional pessimism with perceived stress and psychological maladjustment (e.g., 26, 27), also during the COVID-19 pandemic (28–30). For example, Oh et al. (31) reported that the absence of pessimism than the presence of optimism was more strongly predictive of healthy behaviors that reduce the risk of infection during the COVID-19. The trait-driven effects also are valid among adolescents (32). Thus, optimism and pessimism deserve to be treated independently, which piqued our curiosity about how early adolescents’ dispositional optimism−pessimism determined individual differences in emotional maladjustment amidst the COVID-19 pandemic.

### 1.2 Potential moderation of knowledge about the disease and conflicts with parents

In previous studies, optimism−pessimism has been conventionally treated as dispositional factor conditioning the relationship between environment and individual’s well-being (e.g., 14, 29, 30, 33). Instead, relatively less has been done to investigate the individual differences behind the relationship of adolescents’ dispositional optimism−pessimism with emotional maladjustment. This study endeavored to examine some potential moderators. Research shows that the effects of dispositional optimism−pessimism on well-being are resolved through intrapersonal and interpersonal processes (34). At the intrapersonal end, optimists persist in more engagement, goal-directed behaviors, and problem-solving or emotion-focused coping strategies than pessimists (11). At the interpersonal end, although empirical evidence has been scarce, it has consistently demonstrated that optimists have more positive social interactions and are more ready to seek social support when facing difficulties than pessimists (34). As such, some pandemic-related factors that were at the intrapersonal and interpersonal ends might adjust the relations of early adolescents’ dispositional optimism−pessimism to emotional maladjustment.

At the intrapersonal end, we showed interest in youth’s knowledge about the COVID-19 disease. Youth learned the information about COVID-19 from various channels, especially the internet media (35). The media exposure to the pandemic helps people know the disease and the epidemic situation, but it also might have affected people’s psychological responses with increased anxiety and heightened stress (36–38). It means that knowledge about the disease might affect their understanding or estimation of the severity and controllability of the pandemic, which may in turn affect their emotional and behavioral reactions and self-regulatory strategies (39). What’s more, because of the judgment bias (40), optimism might lead people to underestimate the severity of the pandemic, while pessimism might lead to people’s overestimation. Although having the similar knowledge, pessimists might have behaved more maladjusted responses than optimists did. For example, in a recent study, pessimists, who were better informed than optimists in COVID-19-related knowledge, showed more depression and anxiety than optimists (38). The study asserted that pessimists might be inclined to expose themselves to the media, receiving misinformation along with correct information. Media exposure might confirm to pessimists that the disease is more uncontrollable compared to the reality. Misinformation and false reports, in particular, could contribute to more maladjustment in pessimistic adolescents (38, 41). However, youth with high optimism might show resilience to the negative effect of media exposure. More knowledge about the virus may even help them recognize the pandemic, positively and scientifically engage in precautionary behaviors, and adapt to the hard period (42, 43). Therefore, youth’s knowledge about the disease may interact with their optimism or pessimism to navigate their adjustment.

At the interpersonal end, focuses were placed on youth’s conflicts with their parents during the pandemic. For school-going children and adolescents, the parent−child relationship during the pandemic may be full of challenges (44, 45). Apart from the adjustment of working remotely from home, or waiting for employment (8), many parents also face new demands of monitoring home-based schooling and children’s use of the internet and regulating children’s daily routines. These demands place increased stress on parents and drive parents to monitor, control, or scold their children, which further increases the risk for conflicts between parents and teenagers (46). Adolescents perceived more conflicts with their parents (e.g., 44). Such relational problems per se led to youths’ emotional maladjustment (47), but also in turn interferes with the interpersonal processes of optimism−pessimism on well-being. Optimism reflects one’s self-regulatory resources for coping with problems or stress (11); however, poor interpersonal relationship per se may be translated into stress, and even offset the positive effect of optimism or intensify the negative effect of pessimism. With more strain in the relationship with their parents, youth high in pessimism may be more likely to experience emotional maladjustment. For optimistic youth, it also may be challenging. The protective role of optimistic minds might have been submerged in conflicts with parents, since social connections with others (e.g., peers) have been hindered by the pandemic (7). Therefore, conflicts with parents may mitigate the protective effect of optimism but intensify the negative effect of pessimism on youth’s emotional maladjustment.

### 1.3 The current study

This study had three aims. First, we examined the associations between early adolescents’ dispositional optimism−pessimism and their emotional maladjustment. It was hypothesized that youth with high optimism experienced less emotional maladjustment, while youth with high pessimism suffered more. Second, we investigated the moderating role of youth’s knowledge about the disease. We expected that, from the intrapersonal process, more knowledge about the disease intensified the links of optimism and pessimism with emotional maladjustment. Third, we also investigated the moderating role of youth’s conflicts with their parents during the pandemic. We expected that, from the interpersonal process, more conflicts intensified the link of pessimism with emotional maladjustment but attenuated the link of optimism with emotional maladjustment. Examining these associations could help understand Chinese early adolescents’ individual differences in emotional maladjustment amidst the hard period.

## 2 Methods

### 2.1 Participants and procedure

This survey was conducted online via a Chinese survey website (www.wjx.cn) from March 1 to April 5, 2020. At the beginning of the online questionnaire, an informed consent form was provided. For youth, after the informed consent form was approved by their parents, they received access. After completing the online questionnaire, all participants were sincerely thanked and paid 10 RMB (approximately 1.43 dollars). A total of 3,995 respondents participated. Referring to DeSimone et al.’s (48) recommendations, we deleted the invalid responses (e.g., being rated with the same option on all the scales, unfinished, from an abnormal IP address, or too long or too short a duration to respond). Then, we included the participants aged 10 to 14 years old or in Grade 4 at least, obtaining a final sample of 2,958 early adolescents. Youth were on average 11.66 years old (*SD* = 1.30); half were girls (50.1%). The majority were identified as Han Chinese (96.2%) and from biological-parent families (87.2%). Approximately three quarters of parents’ educational attainment was junior high school and below, 72.8% and 77.9%, respectively, for fathers and mothers. This study was approved by the Ethics Committee of the corresponding author’s institute.

### 2.2 Measures

#### 2.2.1 Emotional maladjustment

Five self-developed items based on panic, helplessness, anxiety, depression, and anger were used to assess adolescents’ emotional maladjustment (e.g., “I feel helpless”). The participants responded to each item on a 5-point Likert scale from 1 (*strongly disagree*) to 5 (*strongly agree*). We calculated the mean scores by averaging the scores of the five items. Higher scores indicate higher levels of emotional maladjustment. The Cronbach’s *α* was .91.

#### 2.2.2 Optimism−pessimism

This study used the LOT-Revised (13) in the Chinese version. The scale has six items related to optimism (3 items, e.g., “In uncertain situations, I usually expect the best”) and pessimism (3 items, e.g., “If something can go wrong for me, it will”). The participants responded to each item on a 5-point Likert scale of varying degrees of agreement or disagreement. The mean scores were calculated by averaging all the corresponding item scores to yield an optimism score and a pessimism score (49). This scale has been validated in Chinese populations (e.g., 50). In this study, the Cronbach’s *α*s for the optimism subscale and the pessimism subscale were acceptable,.71 and.79, respectively.

#### 2.2.3 Knowledge about the disease

Five self-developed items involving typical symptoms, route of transmission, protective measures, cure rate, and mortality rate, were used to assess one’s knowledge about the COVID-19 disease (e.g., “How much do you know …”). Each item was rated on a seven-point Likert scale ranging from 1 (*do not know at all*) to 7 (*completely know*). The internal consistency was .91.

#### 2.2.4 Parent−child conflicts

In this study, youth’s conflicts with their parents were measured by four self-developed items (i.e., “How often do you show anger, quarrel, ignore, or feel an antipathy with your parents?”). The participants were asked to report the current relationships during the pandemic on a 7-point Likert scale, from 1 (*never*) to 7 (*always*). The internal consistency was good (*α* = .88).

### 2.3 Analysis plan

We first calculated the descriptive statistics of all study variables and the bivariate correlations among them. Then, a hierarchical multiple regression model was conducted to examine the study hypotheses. In this model, sex, age, and optimism−pessimism were entered in Step 1; potential moderators (i.e., knowledge about the disease and parent−child conflicts) were entered in Step 2; two-way interaction terms between optimism−pessimism and potential moderators were entered in Step 3; and three-way interaction terms between optimism−pessimism, knowledge about the disease, and parent−child conflicts was entered in Step 4. We centered the values of all variables (except for sex) and then calculated the interaction terms. Once the significant interaction effect was confirmed, *post-hoc* simple slope analyses were conducted (51). All analyses were conducted within SPSS 26.0.

## 3 Results

The means, standard deviations, and correlation matrix of all study variables are presented in **Table 1**. Sex and age were not significantly associated with emotional maladjustment. Optimism was negatively associated with emotional maladjustment (*r* = -.04, *p* = .029), while pessimism was positively associated with emotional maladjustment (*r* = .50, *p* <.001). Knowledge about the disease and parent−child conflicts were negatively associated with each other (*r* = -.12, *p* <.001), while both were positively associated with emotional maladjustment (*r*_knowledge about the disease_ = .07, *p* <.001; *r*_parent−child conflicts_ = .24, *p* <.001).

**Table 1:** Descriptive statistics and correlations between study variables.

	1	2	3	4	5	6	7
1 Sex	–						
2 Age	.02	–					
3 Optimism	.01	-.06^**^	–				
4 Pessimism	-.03	.03	.00	–			
5 Knowledge about the disease	.01	.00	.33^***^	.03	–		
6 Parent−child conflicts	-.02	.06^***^	-.17^***^	.25^***^	-.12^***^	–	
7 Emotional maladjustment	-.02	.00	-.04^*^	.50^***^	.07^***^	.24^***^	–
Mean	–	11.66	3.96	2.24	3.79	2.28	2.20
*SD*	–	1.30	.98	1.01	.92	1.18	1.09

^*^ *p* <.05, ^**^ *p* <.010, ^***^ *p* <.001.

**Figure 1:**
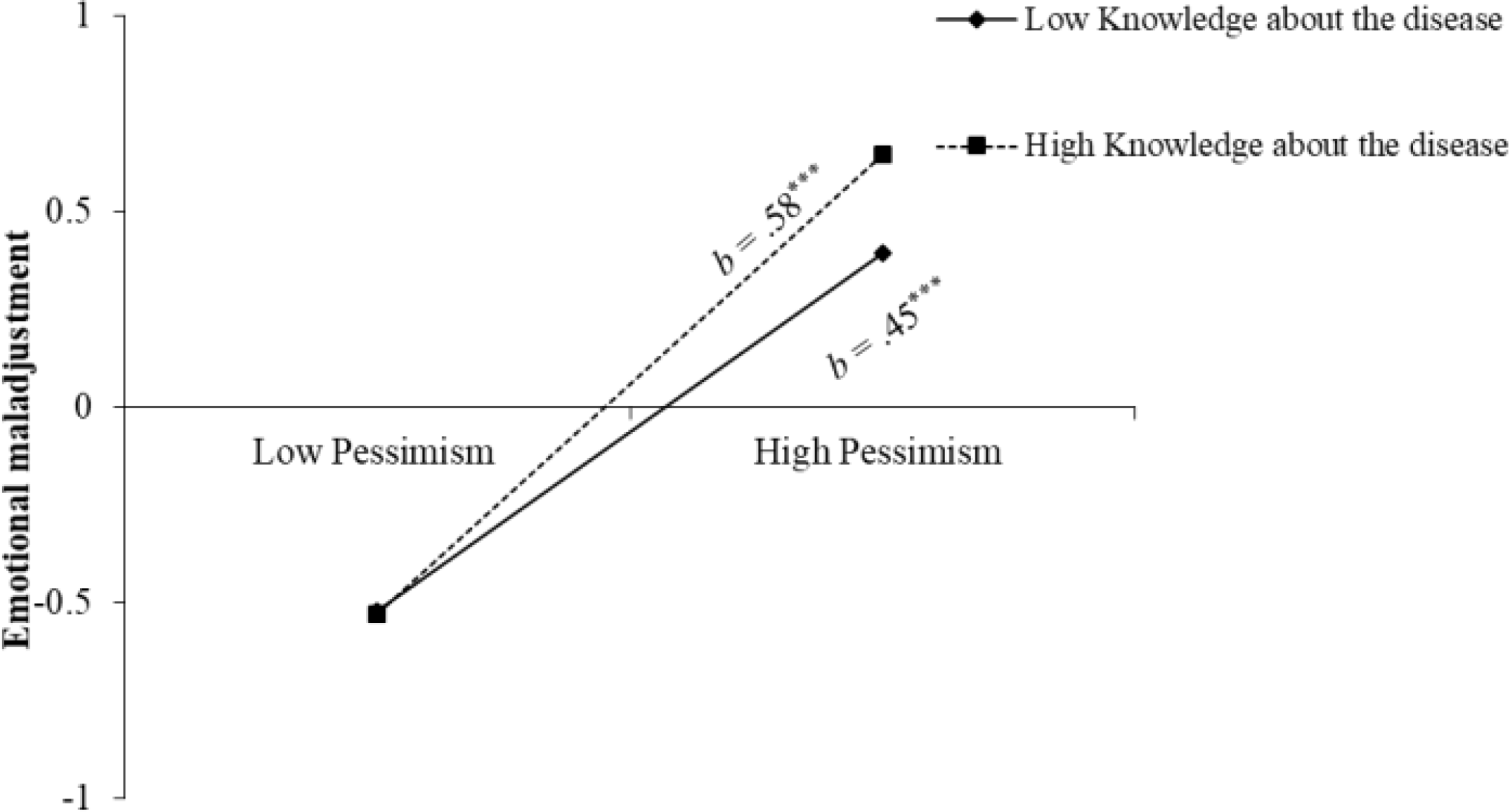
The interaction effect of pemissm and knowledge about the disease. Low and high levels of knowledge about the disease were coded as ± *SD.* ^***^ *p* <.001.

**Figure 2:**
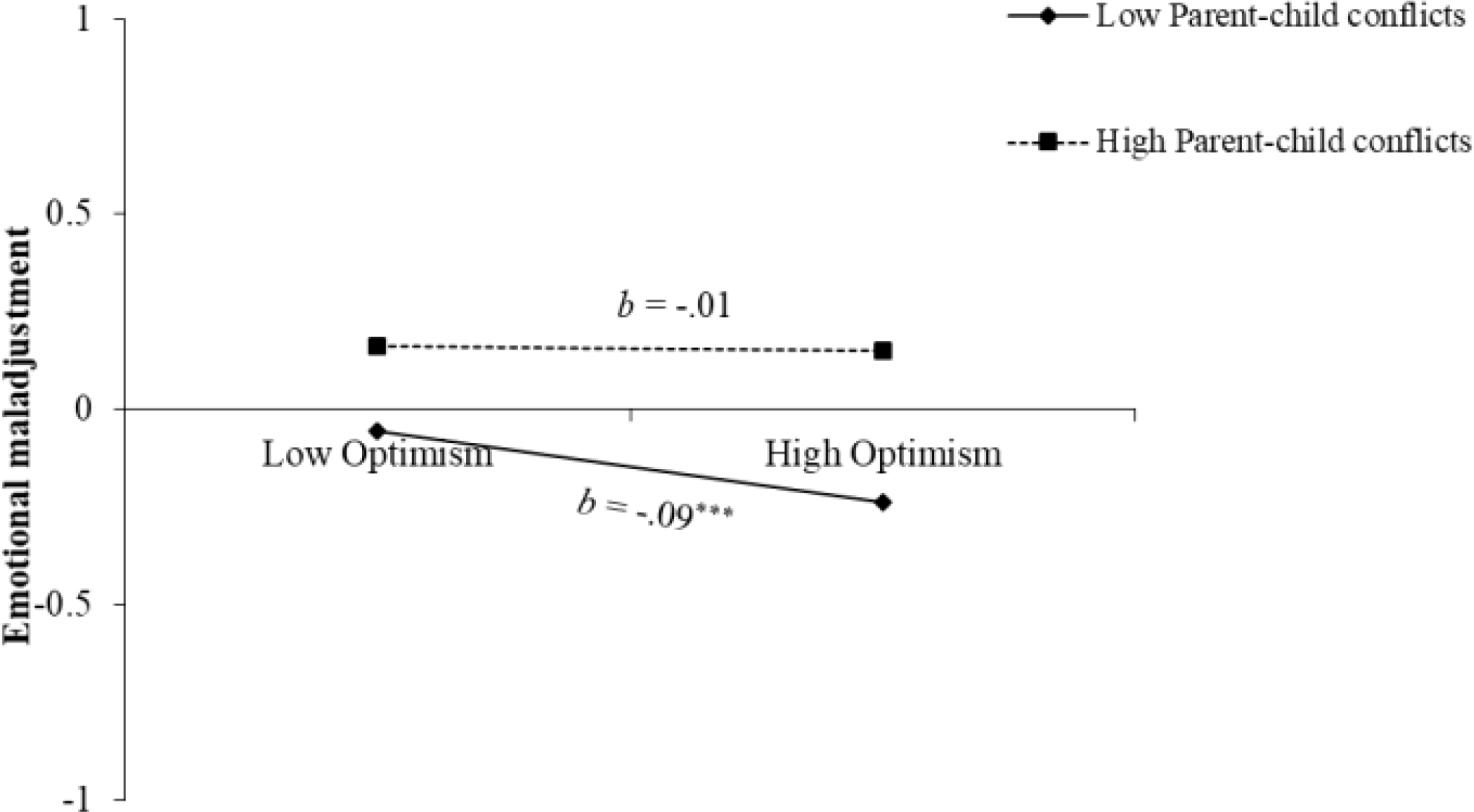
The interaction effect of optimism and parent−child conflicts. Low and high levels of parent−child conflicts were coded as ± *SD.* ^***^ *p* <.001.

The results of the hierarchical multiple regression model are shown in **Table 2**. The first hypothesis was supported. Optimism was negatively associated with emotional maladjustment (*β* = -.04, *p* = .008), while pessimism was positively associated with emotional maladjustment (*β* = .50, *p* <.001). The hypothesis of the moderating role of knowledge about the disease was partially supported, in which the interaction term between pessimism and knowledge about the disease was significant in predicting youth’s emotional maladjustment (*β* = .07, *p* <.001). **Figure 1** depicts the interaction effect of knowledge about the disease, that for youth with more knowledge about the disease, pessimism was associated with more emotional maladjustment (*b*_+1_ *_SD_* = .58, *p* <.001 VS. *b*_-1_ *_SD_* = .45, *p* <.001). The hypothesis of the moderating role of parent−child conflicts also was partly supported, where the interaction term with optimism was significant (*β* = .04, *p* = .013). As depicted in **Figure 2**, compared with youth who experienced more conflicts with their parents (*b* = -.01, *p* = .812), optimism was significantly associated with less emotional maladjustment among youth who experienced fewer conflicts with their parents (*b* = -.09, *p* <.001). Additionally, the effects of the two three-way interaction terms were not significant.

**Table 2:** Hierarchical multiple regression analyses.

	Δ*R* ^2^	*t*	*β*
Step 1	.248^***^		
Sex		-.12	-.00
Age		-1.10	-.02
Optimism		-2.64	-.04^**^
Pessimism		31.10	.50^***^
Step 2	.021^***^		
Knowledge about the disease		5.20	.09^***^
Parent−child conflicts		8.08	.13^***^
Step 3	.006^***^		
Optimism × Knowledge about the disease		-1.77	-.03
Optimism × Parent−child conflicts		2.48	.04^*^
Pessimism × Knowledge about the disease		4.04	.07^***^
Pessimism × Parent−child conflicts		-.34	-.01
Step 4	.000		
Optimism × Knowledge about the disease × Parent−child conflicts		.48	.01
Pessimism × Knowledge about the disease × Parent−child conflicts		1.04	.02

^*^ *p* <.05, ^**^ *p* <.010, ^***^ *p* <.001.

## 4 Discussion

This study paid special attention to the individual differences in early adolescents’ emotional maladjustment during the hard period and examined the trait-driven effects of dispositional optimism−pessimism and the potential adjusting effects of knowledge about the disease from the intrapersonal process and the conflicts with their parents from the interpersonal process. With a large sample of Chinese early adolescents, it was found that dispositional optimism and pessimism were differently associated with emotional maladjustment, and that such associations were adjusted by their knowledge about the disease and conflicts with their parents. These findings help understand early adolescents’ individual differences in emotional maladjustment, support the independent dimensions of optimism and pessimism, and provide some practical implications from both intrapersonal and interpersonal processes, when facing the pandemic or public health crisis contexts in future.

Our findings first support the trait-driven effect of dispositional optimism−pessimism on individual differences in emotional maladjustment (e.g., 32) and echo the proverb that “a pessimist sees a glass half-empty, an optimist sees it half-full”. Amidst the COVID-19 period, youth high in optimism suffered less emotional maladjustment, while those high in pessimism experienced more. In terms of our sample, early adolescents with high optimism might tend to believe the controllability of the disease or to enjoy the period during which they could stay at home with their parents’ companionship. In contrast, when thinking about the situation, pessimistic youth may overestimate the uncontrollability of the pandemic and underestimate their own agency. They might be easily sick of being trapped at home and taking online classes under their parents’ monitoring for a long time. These trait-driven beliefs or reactions contributed to the individual differences. In the face of the pandemic and the anti-epidemic measures in mainland China, the situation for almost all the ordinary people were the same. The individual differences in social adjustment were largely due to these trait-driven beliefs or reactions. Although not examined, the underlying mechanisms might also be related to their attitudes or trust about the government’s anti-epidemic measures, self-regulatory capabilities, coping strategies, and belief- or meaning-making in such threatening situations (e.g., 23, 52–54).

More importantly, by treating optimism and pessimism separately, it was found that the larger effect size and significance of pessimism indicated its higher contribution than that of optimism, although statistically significant associations are shown in both optimism and pessimism with emotional maladjustment. This finding is similar to the study by Scheier and colleagues (18), who emphasized pessimism as a larger predictor of physical health. It also respond Scheier and colleagues’ (18) idea, that the presence of pessimism and the absence of optimism relates differentially to emotional adjustment that arise in reaction to the hard time. The independent effects of optimism and pessimism further support the claim that optimism and pessimism should be regarded as separate entities (16, 18, 21). This also echoes Baumeister et al. (55)’s general principle of psychological phenomena: “bad is stronger than good”. Similarly, in research about the 2003 SARS epidemic, it also was found that pessimism rather than optimism significantly predicted Chinese people’s anxiety (27). This finding hints to us that during hard periods such as SARS or COVID-19 outbreaks, early adolescents high in pessimism deserve more care and support, and parenting practices or other sources of instructions aiming to help adolescents adjust to a more optimistic ideology would be highly rewarded in their future development. It also provides educational directions or further intervention that place a greater weight on lessening pessimism would be promising in promoting adolescents’ adjustment than will those that focus more on promoting optimism (11, 18, 56).

This study also showed that the predisposed effects of optimism and pessimism on emotional maladjustment were conditioned by some pandemic-related intrapersonal and interpersonal factors. At the intrapersonal end, we validated the moderating role of knowledge about the disease. On the one hand, knowledge about the disease per se positively predicts early adolescents’ higher emotional maladjustment (e.g., 36). On the other hand, the link from pessimism to emotional maladjustment was intensified by one’s knowledge about the disease. These findings were inconsistent with previous studies where people’s knowledge about the disease was not related to any behavioral or emotional concerns (57, 58). It may be that participants in these studies were adults who have more recourses, activeness, autonomy, and strategies to manage or control their own lives than the youngsters of this study. More importantly, the results of the present study suggest that the increased negative outcomes may be more exclusive among youth who are more pessimistic (38, 59). From a broader lens, it indirectly supports the previous idea that media exposure can help people know more about the epidemic situation but at the expense of increased psychological deterioration (37). Specifically, the presence of pessimism might lead adolescents to overestimate the severity of pandemic and be more vulnerable in these situations. When felt negative, they easily judged the risk as high and benefits as low (60). And, in the present study, it assessed adolescents’ subjective knowledge about the disease, rather than their answers to some objective questions (e.g., 61), which might have over-inflated the magnitude of the moderating effect. The possible case was that exposure to false or alarming information might also have affected pessimists’ behavioral and emotional responses; that is exposure to alarmist content or conspiracy theories might exacerbate pessimism (39). It also suggests that parents and media should discuss and spread pandemic or epidemic information with early adolescents in a scientific, sensitive, kind, effective, accurate, and age-appropriate way. It could help children through hard periods, especially pessimists (62).

However, in the relation of optimism to emotional maladjustment, we did not find a moderating role of knowledge about the disease. This suggests that the effect of optimism was similar in different levels of obtained knowledge about the disease. This provides proof for the psychological resilience of optimism to media exposure in the pandemic period. Optimistic adolescents were not harmed by gaining more knowledge of the pandemic, while pessimistic adolescents might need guidance to process the knowledge before they expect the worst. It may be that Chinese have a pessimistic bias towards negative events (63), although no examining the cultural difference in this study. Additionally, future research should explore the different styles of optimistic thinking that could be masked by overall resilience. For realistic optimistic adolescents, without proper guidance, learning more about the pandemic may lead them to act with vigilance, which disrupts their routines and social competence (27). On the other hand, for unrealistic optimists, who perceive themselves to be safer than others in the same circumstances, learning more knowledge about the pandemic is likely to be helpful (43, 64). Especially considering evidence that unrealistic optimists are more likely to behave against the recommendations from health care professionals, learning more about the pandemic serves as a protective factor for their physical and psychological health (64, 65).

At the interpersonal end, our findings revealed that parent−child conflicts adjusted the relation of optimism to emotional maladjustment. Specifically, the positivity of optimism when facing hardships was buried, if the adolescents experienced more conflicts with their parents. This finding suggests the significance of the parent−child relationship (66), since during the pandemic period, other significant close relationships, such as those with close friends were disrupted, and children were more dependent on their relationships with their parents (45, 67). Unfortunately, the pandemic and related measures have made parenting and relationships more challenging, and conflicts occur frequently (44, 46). Conflicts with their parents were impactful on adolescents’ emotional adjustment (e.g., 58). The positivity of optimistic minds was compromised. This may reflect the destructiveness of the collision between early adolescents’ eagerness for autonomy from their parents and parents’ frequent monitoring during the pandemic (68). However, only the presence of optimism on emotional maladjustment was moderated by parent−child conflicts, instead of the absence of pessimism, again suggesting the separability between optimism and pessimism. Parent−child conflicts did not moderate the link from pessimism to emotional maladjustment, which may be due to the powerful negativity of pessimism per se on psychological health (18). Since adolescents high in pessimism were already emotionally harmed, as parent−child conflicts escalated, the additional layer of difficulty was rather marginal. Additionally, adolescents high in pessimism are more likely to experience relational problems (34), so the synergistic effect of parent−child conflicts with dispositional pessimism on adolescents’ maladjustment was not evidenced. These findings highlight that parents’ efforts to maintain daily interactions in a kind, friendly, and supportive manner with their adolescents would be rewarded during the pandemic or other possible public health crisis contexts in future.

Several limitations of this study should be discussed. First, the design of our study was cross-sectional, which was unable to reveal the causal associations among study variables. For example, the more positive youth were in responding to the pandemic, the more accurate and effective they might have acquired knowledge about the diseases (53). Second, we used online questionnaires and nonrandom sampling, which may have affected the representativeness and reliability of the results. Adolescents who refused to participant into our survey might have been suffering the hard period. And, the use of self-reports to measure emotional adjustment and parent interactions might have introduced biases, such as social desirability or recall issues. Third, except for dispositional optimism−pessimism, other variables were measured by self-developed tools, which may have affected the reliability of our results. For example, knowledge about the disease was limited in its ability to distinguish between types of knowledge (e.g., factual knowledge versus misunderstandings or misinformation). It might also limit the comparison with other related research. Last, this study was from China. The cultural specificity in optimism and pessimism in Chinese society may affect the generalization of the results and comparison with other studies in Western society (69). Additionally, this survey was started at the end of the Level I emergency response to COVID-19 (which began on January 23, 2020 and turned into Level II in late February in succession). That is, some families from some provinces had ended their home confinement or had become accustomed to staying at home, so the negative effect of the pandemic might be less salient in this context, compared to studies in other countries.

## Data Availability

The data underlying this manuscript will be shared on reasonable request to the corresponding author.
